# Plant Non-Coding RNAs: Origin, Biogenesis, Mode of Action and Their Roles in Abiotic Stress

**DOI:** 10.3390/ijms21218401

**Published:** 2020-11-09

**Authors:** Joram Kiriga Waititu, Chunyi Zhang, Jun Liu, Huan Wang

**Affiliations:** 1 Biotechnology Research Institute, Chinese Academy of Agricultural Sciences, Beijing 100081, China; joram.kiriga@gmail.com (J.K.W.); zhangchunyi@caas.cn (C.Z.); 2 National Key Facility for Crop Resources and Genetic Improvement, Institute of Crop Science, Chinese Academy of Agricultural Sciences, Beijing 100081, China; liujun@caas.cn

**Keywords:** non-coding RNA, long non-coding RNA, abiotic stress, transcriptional, biogenesis

## Abstract

As sessile species, plants have to deal with the rapidly changing environment. In response to these environmental conditions, plants employ a plethora of response mechanisms that provide broad phenotypic plasticity to allow the fine-tuning of the external cues related reactions. Molecular biology has been transformed by the major breakthroughs in high-throughput transcriptome sequencing and expression analysis using next-generation sequencing (NGS) technologies. These innovations have provided substantial progress in the identification of genomic regions as well as underlying basis influencing transcriptional and post-transcriptional regulation of abiotic stress response. Non-coding RNAs (ncRNAs), particularly microRNAs (miRNAs), short interfering RNAs (siRNAs), and long non-coding RNAs (lncRNAs), have emerged as essential regulators of plants abiotic stress response. However, shared traits in the biogenesis of ncRNAs and the coordinated cross-talk among ncRNAs mechanisms contribute to the complexity of these molecules and might play an essential part in regulating stress responses. Herein, we highlight the current knowledge of plant microRNAs, siRNAs, and lncRNAs, focusing on their origin, biogenesis, modes of action, and fundamental roles in plant response to abiotic stresses.

## 1. Introduction

Plants are sessile species that cannot evade stress but require mechanisms of avoidance and/or tolerance for survival when exposed to ever-changing environmental conditions. Abiotic stresses including cold, heat, hypoxia, drought, nutrient deficiency and salinity affect plant growth and productivity, resulting in substantial loss of yields [1]. Also, the ever-increasing global population, decreasing arable land due to rapid urbanization, significant changes in soil quality and global warming are all projected to exacerbate food security issues further [2].

Non-coding RNAs (ncRNAs) have risen dramatically in recent years as essential bioactive molecules contributing to genomic and phenotypic diversities. While up to 90% of the eukaryotic genome is transcribed into RNA, only about 2% of transcribed RNAs generate protein products [3,4]. The remaining transcriptome comprises ncRNAs transcripts, which were previously considered transcriptional noise owing to the absence of protein-coding ability and poorly conserved sequences [3,5]. However, high-throughput sequencing analysis and experimental validation have confirmed the involvement of ncRNAs in a myriad of distinct gene regulatory levels, including epigenetic, transcriptional, and post-transcriptional [6,7,8]. Thus, the advancement of effective next-generation sequencing (NGS) technologies [9,10] in conjunction with homology-based and/or experimental approaches has been vital to discovering ncRNAs in plants.

**Figure 1:**
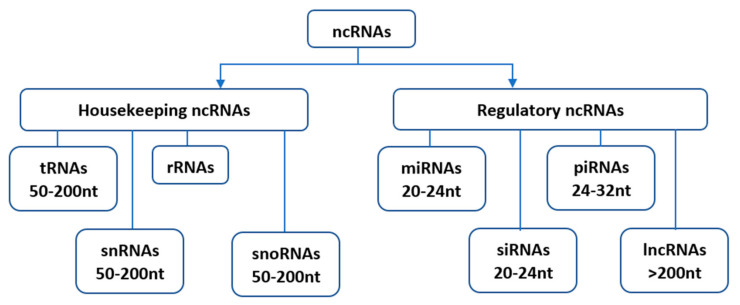
Classification of non-coding RNAs (ncRNAs). Housekeeping ncRNAs include; tRNAs- transfer RNAs, snRNAs-small nuclear RNAs, rRNAs-ribosomal RNAs, snoRNAs-small nucleolar RNAs. The regulatory ncRNAs consist of miRNAs-microRNAs, siRNAs-short interfering RNAs, piRNAs-piwi-interacting RNAs, and lncRNAs-long non-coding RNAs.

Since their initial discovery, ncRNAs have been categorized into several distinct classes based on their origin, biogenesis, and mechanism of action. Housekeeping and regulatory ncRNAs are the major groups of ncRNA transcripts. The housekeeping ncRNAs primarily deals with cellular and ribosomal functions. They include transfer RNAs (tRNAs), small nuclear RNAs (snRNAs), ribosomal RNAs (rRNAs), and small nucleolar RNAs (snoRNAs) (Figure 1). On the other hand, the regulatory ncRNAs consist of microRNAs (miRNAs), short interfering RNAs (siRNAs), piwi-interacting RNAs (piRNAs), and long non-coding RNAs (lncRNAs) [11], (Figure 1). These regulatory ncRNAs are transcribed from DNA but lack the ability to translate into proteins. [7,12]. Nevertheless, they perform a myriad of vital functions in plant growth, development, and abiotic stress responses at transcription and post-transcription levels [13].

Over the past decades, fundamental advancement has been achieved in unfolding the dynamic and complicated mechanisms underlying plant response to environmental stresses. Considerable effort has been channelled towards identifying stress-responsive genes as well as their respective protein interaction networks. This led to the observation that plant stress response depends on the accurate expression and regulation of genes achieved via multiple mechanisms at various levels like transcriptional and post-transcriptional regulations [14,15]. However, despite substantial scientific research that have extensively focused on the regulatory mechanisms of coding proteins, recent findings have pinpointed the essential functions of ncRNAs in controlling transcriptional and post-transcriptional gene expression levels during plant developmental and environmental stress responses [16]. In this review, we summarize the current tremendous progress on ncRNAs molecules from miRNAs, siRNAs to lncRNAs with an extensive emphasis on their origin, biogenesis, modes of action, and diverse roles orchestrating plant abiotic stress responses.

## 2. Origin and Biogenesis of Non-Coding RNAs

ncRNAs are diverse in both their origin and functions [17]. Duplication, evolution from existing transposable elements (TEs), random hairpin structures, pseudogenization of protein-coding sequences, replication of RNA viruses, and double-stranded RNAs (dsRNAs) from heterochromatin regions, and DNA repeats [18,19,20,21] are the leading theories for the origin of various ncRNAs such as miRNAs, siRNAs, piRNAs, and lncRNAs.

**Figure 2:**
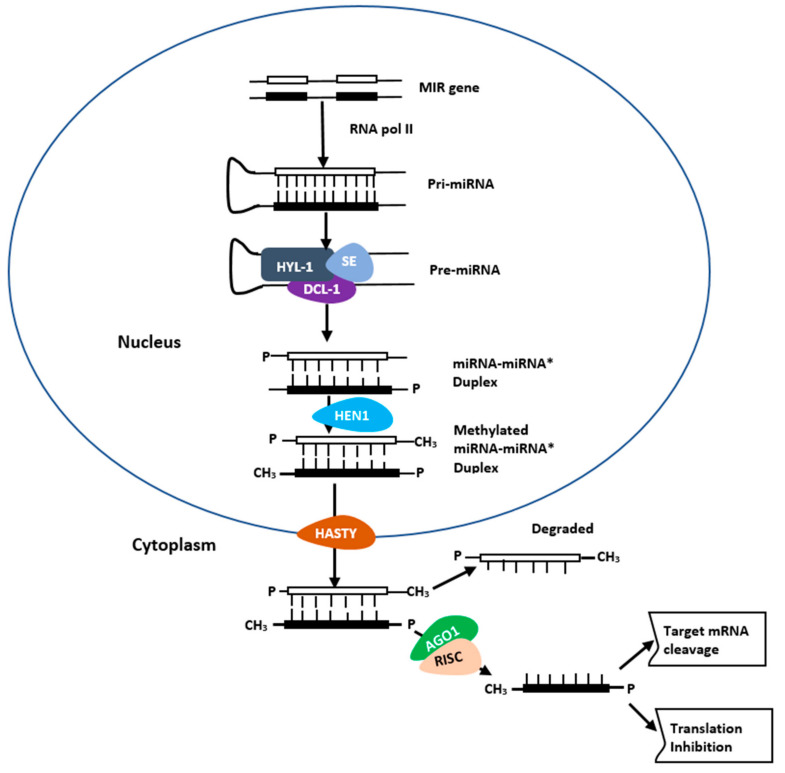
Biogenesis of plant miRNAs. In the nucleus, MIR genes are processed to miRNA/miRNA* duplexes through the action of Pol II, DCL1, SE, HYL1, HEN1, and HASTY. In the cytoplasm, the duplex is incorporated with RISC-AGO complex to which guides it towards the target, resulting in suppression of the translation and cleavage of mRNAs.

MicroRNAs are small but fundamental molecules which are predominantly 20–22 nt in length [22]. They are synthesized from miRNA genes (MIR genes), which are processed by RNA polymerase II (RNA pol II), RNase III Dicer-like protein 1 (DCL1) [23,24,25] (Figure 2). HASTY (HST) transports the resulting miRNA–miRNA* duplex to the cytoplasm where it is integrated into the RNA-induced silencing complex (RISC) containing ARGONAUTE 1 (AGO1) proteins that control gene silencing by mRNA cleavage and translational repression [26,27,28], (Figure 2).

**Figure 3:**
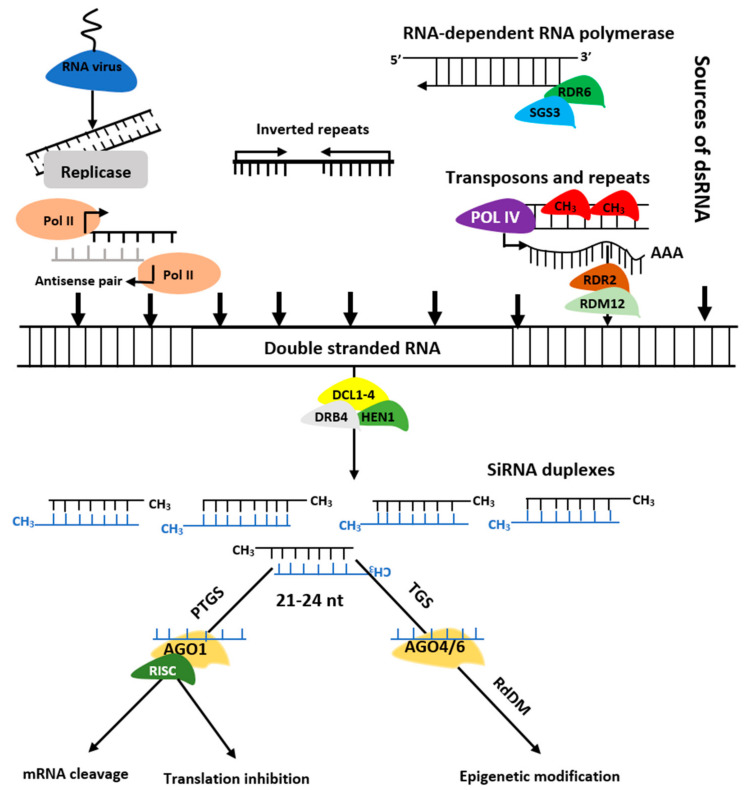
Biogenesis of siRNAs. The double stranded RNA (dsRNAs) is transformed into siRNAs by DCL, HEN1, and DRB. The RISC-AGO complex then guides the selected strands of siRNA duplexes to post transcription gene silencing (PTGS) or transcription gene silencing (TGS).

The siRNAs are grouped into trans-acting siRNAs (tasiRNA), heterochromatic siRNAs (hc-siRNA), and natural antisense siRNAs (nat-siRNAs) depending on the mode of action as well as biogenesis. tasiRNAs have a regulatory mechanism similar to miRNAs, and they act in *cis* and operate at loci independent from their biogenesis site. At the same time, hc-siRNA works effectively in *cis* within their source or homologous regions as their origins align with their targets [29]. However, nat-siRNAs are categorized into cis-nat-siRNAs and trans-nat-siRNAs. The generation of the former involves transcription of two RNAs from opposite strands of the same loci. Simultaneously, the latter involves RNA transcripts hailing from multiple loci [30]. The siRNAs biogenesis involves dsRNAs, DCL enzymes activities, and RISC containing AGO protein that controls target regulation at post-transcription or transcriptional level [31] (Figure 3). Biogenesis of ta-siRNA is dependent on SUPPRESSOR OF GENE SILENCING3 (SGS3), RNA-dependent RNA polymerases 6 (RDR6), and DCL4 [32,33] while that of nat-siRNAs depends on SGS3, RDR6, DCL1, DCL2, and a plant-specific RNA polymerase, NRPD1A [34]. Similarly, hc-siRNAs are generated by Pol IV, DCL3, RDR2, 5S rRNA genes, and NRPD1A [35,36].

**Figure 4:**
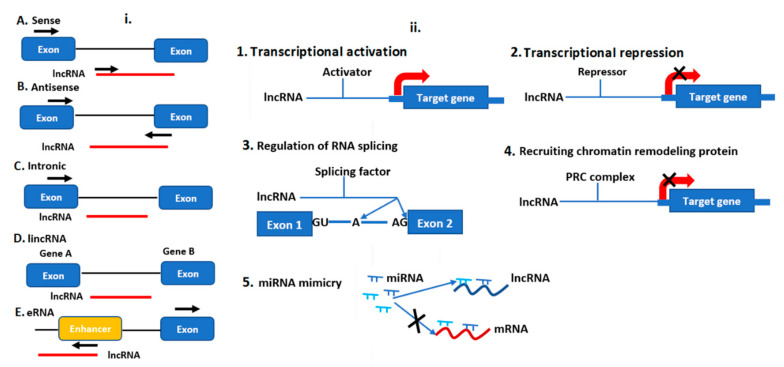
The biogenesis of long-non-coding RNAs (lncRNAs) and their gene regulation mechanisms in plants. (**i**) The transcripts of lncRNA (red box) are classified on the basis of their genomic location and in relation to the nearest gene (blue box): (**A**) sense lncRNAs are transcribed on the same strand as an exon; (**B**) antisense lncRNAs are transcribed on the opposite strand of an exon; (**C**) intronic lncRNAs are transcribed on the intron; (**D**) intergenic lncRNAs are located between two distinct genes; (**E**) enhancer lncRNAs emerge from an enhancer region of protein-coding genes. (**ii**) The gene regulation pathways induced in plants by lncRNAs. LncRNAs regulate gene expression either by: (**1**) interacting with transcriptional activator leading to gene activation; (**2**) interacting with transcriptional repressor thereby suppressing transcription; (**3**) controlling RNA splicing by interacting with splicing factor or binding premRNA splicing junction; (**4**) recruiting chromatin remodeling complex such as PRC to regulate gene expression in the promoter region; (**5**) LncRNA mimics miRNAs by occupying their target sites on the mRNA.

Long non-coding RNAs are transcripts that exceed more than 200 base pair (bp) in length and cannot be translated into protein [37]. They are classified into cis-natural antisense (cis-NATs), trans-natural antisense transcripts (trans-NATs), and pseudogenes [38], sense or antisense (strand of origin) [39], divergent, or convergent (orientation of transcription), and as intronic or intergenic (location) [40] (Figure 4). They are transcribed by RNA pol II, III, IV, and V and control target regulation via multiple ways, including chromatin remodeling, transcriptional repression, mimicry, RNA splicing and transcriptional enhancer (Figure 4).

## 3. Impact of Non-Coding RNAs on Plant Gene Regulation

sRNAs such as miRNAs and siRNAs regulate gene expression through a multitude of methods, including (1) cleavage of target mRNA, (2) translational repression, and (3) transcriptional silencing [38,41,42,43]. Similarly, lncRNAs control gene expression through the hijacking of protein and miRNAs, regulating mRNA stability and translation, and changing chromatin status [44].

### 3.1. Cleavage of Target Transcripts by miRNAs and siRNAs

The sequence-specific cleavage silencing mechanism depends on the binding of the miRNAs and/or siRNAs to the complementary sites in target mRNA molecules [41,45]. For miRNAs, the MIR genes are processed by DCL1, HYL1 and SE complex [20,24,25] into mature miRNA–miRNA* duplex and transported by HASTY protein to the cytoplasm where it is integrated with RISC containing AGO1. The RISC-AGO1 complex binds to the complementary sites of sense sequence on its targeted RNA transcript and degrade it. In contrast, the antisense strand of miRNAs remains in the RISC [46]. In siRNAs, one strand which is integrated with RISC-AGO1 or AGO7 complex is guided to cleave the transcripts of the target gene at 10–11 nt upstream of the 5′ end of the antisense strand [47]. The EXONUCLEASE 4 (XRN4) enzyme then degrade both the 3′ and 5′ cleaved fragments [48,49]. Recent studies have reported the RNA binding and target slicer activities of AGO2, AGO4, and AGO10 in plants, indicating the complexity of the sRNA-induced gene silencing mechanism [50,51].

### 3.2. miRNAs and siRNAs Induced Translational Inhibition of Target Genes

In plants, AGO1 and AGO10 stimulate translational inhibition mechanism [52,53] by imperfectly pairing sRNAs with the target mRNA [45,54]. However, the inhibition rate is extremely dependent on the number of miRNA binding sites [55,56]. The RISC-AGO1 complex suppresses translation by binding to the 5‘untranslated region (UTR) or open reading frame (ORF) of the target gene, thereby restricting ribosome recruitment or movement [57]. This translation inhibition process is regulated by other factors such as ALTERED MERISTEM PROGRAM 1 (AMP1), VARICOSE (VCS), GW-repeat protein, and microtubule enzyme KATANIN (KTN1) [58,59]. Despite the involvement of both AGO1 and AGO10, the specific function of each AGO gene in this inhibition process is still not clear [58,59]. Further research is still needed to fully understand the underlying mechanism of sRNAs mediated translation repression and how the repressed target mRNAs escape endonucleolytic cleavage.

### 3.3. miRNA and siRNA-Directed DNA Methylation

The DCL family has multiple copies in *Arabidopsis*, which contribute to the biogenesis of divergent lengths of sRNA. DCL1 is involved in the conversion of partially paired dsRNA precursors into 21-nt mature miRNAs [60]. On the other hand, DCL2 and DCL44 initiate the generation of 20–22nt siRNAs from perfect complementary dsRNA precursors [61,62]. DCL3 produces 24-nt siRNAs (hc-siRNA) that typically silence gene expression via the RdDM pathway [63,64]. These hc-siRNAs are transcribed at the heterochromatic regions where they stimulate cytosine methylation in the sequence contexts of CG, CHG, and CHH, in cis [65,66]. The DCL3-dependent miRNAs integrate with AGO4 forming a complex which repress gene silencing by cytosine and histone methylation [67]. However, the de novo hc-siRNA-induced RdDM process integrates the activities of RDR, DCL, AGO, and Pol IV and V which transcribe the double-stranded precursors and promote methylation at the target sites respectively [64]. Therefore, DNA and lysine methylation at the ninth position of histone H3 (H3K9) induces systemic silencing [66], while the hc-siRNA/AGO4 RISC guides DNA and H3K9 methyltransferases to the target sequence for transcriptional gene silencing [65,66].

### 3.4. Gene Expression Regulation by lncRNAs

Long non-coding RNAs are intermediates between ribonucleic acids and proteins. They regulate gene expression as transcriptional activators or repressors [68], though the molecular basis of how they do so is poorly understood in plants. In this section, we only discussed the lncRNAs gene regulation mechanisms, which have been characterized so far to alter transcriptional and post-transcriptional expression levels in plants.

Plant lncRNAs function in *cis* and *trans*. The *cis*-acting lncRNAs function near their synthesis sites and operate directly on the local nucleotide sequences or chromosome regions on one or more contiguous genes. The *trans*-acting lncRNAs, on the other hand, disperse from the synthesis site and can function on several genes at great distances, including on different chromosomes [69]. Moreover, lncRNAs can also be transcribed as sRNAs precursors [70]. For instance, a subset of lncRNAs can form double-stranded RNA duplexes with Natural Antisense Transcripts (NAT) to produce sRNAs which carry out their regulatory functions. In *Arabidopsis*, the Rab2-like gene and a pentatricopeptide repeat gene generated a NAT pair from their complementary region with an endogenous siRNA [71]. LncRNAs can serve as miRNA target mimics where the lncRNAs competes with the target mRNA for miRNA binding, thereby blocking the action of the miRNA and relieving repression of its target gene [72]. In *Arabidopsis* under phosphate deficiency (P) conditions, a lncRNA called *Induced by Phosphate Starvation 1* (IPS1) was reported to mimic miRNA399 [73,74]. Moreover, lncRNAs often functions through protein-protein interactions, modification, localization [75,76,77], and via epigenetic regulation mechanisms such as DNA methylation, histone modification, and chromatin remodeling [78]. Like DNA methylation, lncRNAs have strand-specific distribution and expression patterns, which qualifies them to be ideal regulators of DNA methylation. The COOLAIR and COLDAIR lncRNAs repress the expression of FLOWERING LOCUS C (FLC) via chromatin modification during vernalization [79].

## 4. Function of ncRNAs in Response to Abiotic Stress in Plants

Plants have developed various mechanisms to adjust their growth and development during environmental stress conditions. Multiple research findings have recently shown the differential expression of ncRNAs in such unfavourable conditions [80,81]. These ncRNAs either control gene expression in related cellular networks or function directly in response to stress [82]. In this section, we focused on the roles of miRNAs, siRNAs, and lncRNAs on abiotic stress response in plants.

### 4.1. Involvement of miRNAs in Abiotic Stress Response

miRNAs have become new targets for improving plant productivity and abiotic stress tolerance due to the broad functions of their targets [83]. The regulation of miRNAs appears to rely on their roles on the abiotic stress response. Stress-up-regulated miRNAs might down-regulate their target genes, which might be negative regulators of stress tolerance (e.g., stress-responsive gene repressors) [84]. Moreover, the down-regulation of miRNAs under stress might accumulate their target gene mRNAs, which might positively regulate stress tolerance [84]. In this section, we focused on the common significant roles of miRNAs and difficulties encountered to systematically characterize these miRNAs in response to abiotic stress in plants. 

**Figure 5:**
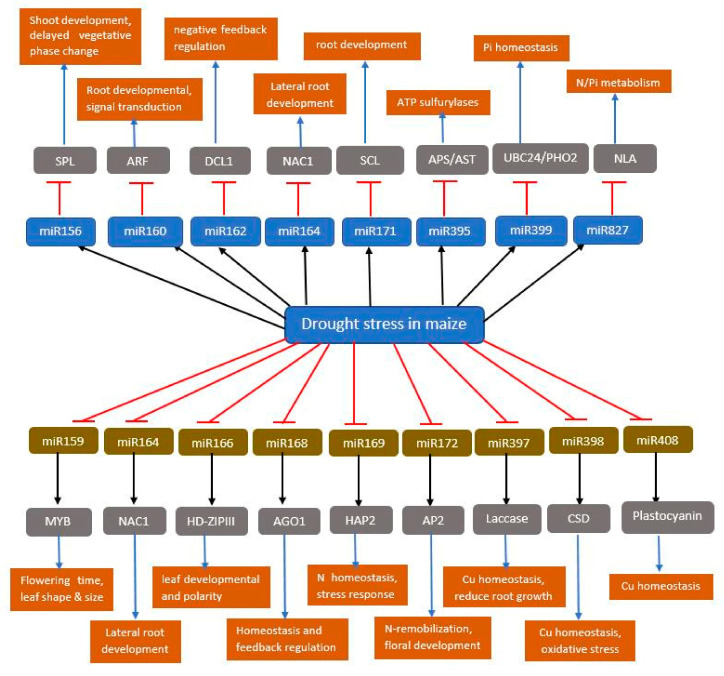
The miRNAs-guided target gene regulations under drought stress in maize. miRNAs that are positively regulated by drought stress (black arrow and blue rectangle) target negative regulators (top gray rectangle) of stress tolerance for enhanced suppression (red blunt arrow) of target gene products. By contrast, miRNAs that are suppressed by drought stress (red blunt arrow and gold rectangle) likely target positive regulators (bottom gray rectangle) of stress tolerance resulting in the accumulation of gene products (orange rectangle) which regulate drought stress response. SPL, sporocyteless; ARF, auxin response factor; DCL1, dicer like protein; NAC1, no apical meristem; SCL, scarecrow-like 3; APS, ATP sulfurylase; UBC24/PHO2, ubiquitin-conjugating Enzyme E2/phosphate 2; NLA, nitrogen limitation adaptation; MYB, myeloblastosis; HD-ZIP III, homeodomain-leucine zipper 3; HAP2, heme activator protein 2; AP2, apetala 2; CSD, copper/zinc superoxide dismutase.

The expression of miRNAs is enhanced or suppressed in response to distinct abiotic stress. MiR393, miR397b, and miR402 were up-regulated in *Arabidopsis*, while miR319c and miR389a were down-regulated under dehydration stress [15]. Similarly, under drought stress in rice at the seedling stage, 17 and 16 miRNAs were suppressed and enhanced, respectively [85]. In maize under drought stress conditions, 8 and 13 miRNAs were up-regulated and down-regulated respectively in the leaves while 7 miRNAs were up-regulated and another 7 down-regulated in the roots [86]. Drought stress, on the other hand, increased the expression of miR156, miR160, miR162, miR164, and miR171, leading to a decrease in the expression of their target genes (Figure 5). miR160 target auxin response factors (ARFs) genes. ARF16 is reportedly involved in the development of root cap cells, while ARF17 acts as a nearly auxin-response gene regulator. Down-regulation of ARF16 and ARF17 will repress plant growth and thereby increase the resistance of maize to drought stress. Likewise, down-regulation of miR169 (Figure 5) led to the accumulation of nuclear factor Y (NFY), which regulates the expression of nitrate transporters, *AtNRT2.1* and *AtNRT1.1* [87].

**Figure 6:**
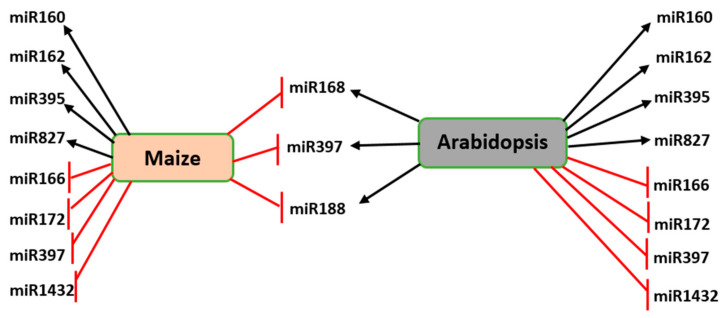
Differential expression of commonly expressed miRNAs in maize and *Arabidopsis* during the drought stress response. Expression of miR168, miR397, and miR188 is down-regulated in maize but up-regulated in *Arabidopsis*. A red blunt arrow indicates a decrease while the black arrow represents an increase in the expression of the corresponding miRNA.

Moreover, one miRNA maybe both down-regulated and up-regulated in the same abiotic stress response in one plant. The expression of miR398a/b in the *Medicago truncatula* was found to be enhanced and repressed in response to drought stress according to different reports [88]. Similarly, miR156, miR164, and miR171 exhibited discrete regulatory trends under drought stress in maize [86], while miR156 and miR171 exhibited contrary regulations patterns in two individual studies of rice under drought stress [89,90]. Additionally, contrasting miRNA expression during the same abiotic stress conditions has been reported in two different plant species. The expression of miR159 in maize and *Arabidopsis* was suppressed in response to drought stress, but enhanced in rice and wheat, while miR399 was up-regulated in maize and wheat, but down-regulated in rice. miR168, miR397, and miR188 were all enhanced in *Arabidopsis* and suppressed in maize (Figure 6) [86,91,92]. More research is required to explain the discrepancy in the expression of such miRNAs, which might be due to differences in the severity of abiotic stress and/or other plant growth conditions.

Some miRNAs are evolutionarily preserved among plant species in stress adaptation. Several miRNAs such as miR169, miR171, miR395, miR397, miR398, miR399, miR408, and miR827, were all up-regulated in both *Arabidopsis* and maize in response to nitrogen (N) deficiency [93,94]. Similarly, miR160, miR162, miR395, miR827, were all up-regulated, while miR166, miR172, miR397, miR827, and miR1432 were all down-regulated in maize, *Arabidopsis*, rice, and wheat in response to drought stress (Figure 6) [86,90,91,92]. Conservation of these miRNAs suggests they have preserved biological functions, but miR171 exhibited distinct patterns of expression under drought stress in maize and rice [86,89,90]. Thus, further research is required to investigate what causes such variations in such comparable situations.

**Figure 7:**
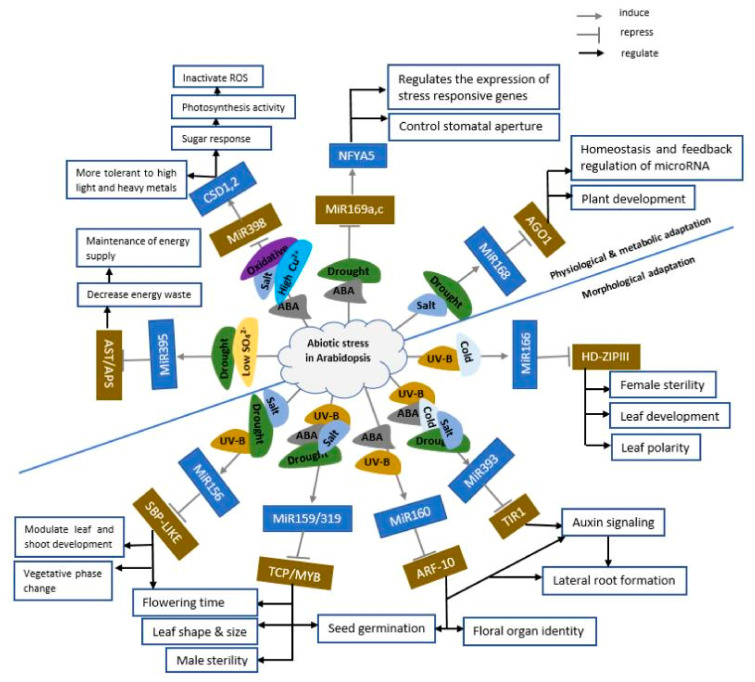
Regulatory network of miRNAs in *Arabidopsis* under abiotic stress. The proposed network describes the molecular mechanisms underlying the response of *Arabidopsis* plants to various abiotic stresses. The network is solely based on alterations in miRNA expression patterns and subsequent target transcripts in stressed plants.

Interestingly, some miRNAs are generally responsive to various abiotic stresses. In *Arabidopsis*, the expression of miR398 was inhibited by oxidative stress, salt, ABA, and high Cu^2+^, while miR169a and miR169c were inhibited by drought and ABA stress [95] (Figure 7). In comparison, the expression of miR393 was induced in *Arabidopsis* by salt, drought, cold, ABA, and UV-B stresses (Figure 7). Also, some miRNAs respond to certain abiotic stresses differently. In *Arabidopsis*, miR169 was induced by salt, cold, and UVB irradiation but suppressed by drought, heat, and ABA treatment [95].

Several studies have shown that certain miRNAs display tissue-dependent expression patterns. During drought stress, more miRNAs were expressed in roots than leaves of peach, indicating that root is more susceptible to drought than leaf tissue [96]. In maize, miR166 was down-regulated in leaves and up-regulated in roots under drought stress [86], while in wheat, six miRNAs (miR159, miR172, miR319, miR399, miR528, and miR4393) were induced in leaves but inhibited in roots under drought stress conditions [91]. Likewise, four miRNAs (hvu-miR156a, hvu-miR166, hvu-miR171, and hvu-miR408) were induced in barley leaves by drought stress, but hvu-miR166 was suppressed in barley roots while the remaining three were unchanged [97].

The treatment method applied for abiotic stresses in plants also contributes to the variation in the expression patterns of the same miRNAs [88]. For instance, polyethylene glycol (PEG) simulated drought stress differed significantly from the actual field drought [98]. In maize, the expression of miR398 was induced by PEG treatment but down-regulated under natural drought conditions [99,100]. PEG treatment up-regulated miR159 and miR166 in maize leaves while field simulated drought down-regulated both miRNAs [100,101,102]. Moderate and severe filed drought down-regulated and up-regulated miR156, respectively, while 16 and 24 h PEG simulated treatment up-regulated and down-regulated miR156, respectively [99,102]. Field simulated drought stress up-regulated miR399 in both leaves and roots, but PEG simulated treatment down-regulated expression of miR399 [99]. These findings suggest ambiguity between field and PEG simulated drought on the expression of miRNAs.

### 4.2. Roles of siRNAs in Abiotic Stress Response in Plants

Recently, the involvement of siRNAs in plant abiotic stress responses has been explored [15]. In *Arabidopsis*, the pairing of the nat-siRNA SRO5 and Pyrroline-5-carboxylate dehydrogenase (P5CDH) regulated proline metabolism, thereby mitigating reactive oxygen species (ROS) induced by high salt stress [34]. The levels of TAS1, TAS2, and TAS3 tasiRNAs were significantly elevated in hypoxia-treated *Arabidopsis* samples indicating their function in stress response [103,104], (Table 1). These variations in tasiRNA levels have been shown to correlate with the TAS targeting levels of miRNAs (miR173 and miR390) [103]. Pentatricopeptide repeat (PPR) mRNAs are the targets of TAS2, and down-regulation of these targets are associated with the defense of mitochondria during hypoxic [105]. The HEAT-INDUCED TAS1 TARGET1 (HTT1) and HTT2 are TAS1 targets that are normally induced by heat stress conditions in *Arabidopsis* [106]. Over-expression of TAS1 reduces the HTT gene expression levels, thereby causing weaker thermotolerance, while over-expression of HTT genes up-regulates HSF genes, thereby increasing thermotolerance [107]. Transcription of HTT genes was induced in heat-tolerant transgenic plants over-expressing the HSFA1a gene. This HSFA1a gene was reported to activate the expression of HTT by binding to their promoters [107]. In *Arabidopsis*, HTT1 was found to interact with HSP70-14 and HSP40. Collectively, these results suggest that HTT1 is a cofactor of HSP70-14 complexes, mediates TAS1a-targeted thermotolerance pathways, and is triggered by HSFA1a [107]]. On the other hand, miR828 regulated ta-siRNAs (TAS4) transcript is critical for the biosynthesis of anthocyanins in response to phosphate deficiency in *Arabidopsis* [108,109]. The induction of TAS4-siR81(-) led to the accumulation of anthocyanin in nitrogen deficiency conditions [109], (Table 1). TAS4-siR81(-) targets MYB transcripts which are vital for the biosynthesis of anthocyanin.

In a recent study, a copia-type retrotransposon called ONSEN was activated in *Arabidopsis* seedlings under heat stress conditions [110]. In addition, new ONSEN insertions were observed to occur in stressed plants deficient in siRNA biogenesis via retrotransposition. These insertions were passed on to the next generation, and they conferred heat responsiveness to nearby genes [110,111,112], (Table 1). Thus, the siRNA-directed pathway plays a significant role in controlling transgenerational retrotransposition triggered by abiotic stress. A research study in Chinese cabbage suggested that several chloroplasts small RNAs (csRNAs) and nat-siRNAs were responsive to heat stress [113,114]. Differential expression analysis in Brassica rapa showed that nat-siRNAs derived from 12 cis-NATs were susceptible to heat stress, with the transcripts producing heat-responsive nat-siRNAs being up-regulated while those transcripts from the opposite strands of the same loci being down-regulated under heat stress [114], (Table 1). Similarly, a *Craterostigma plantagineum* dehydration-related ABA-inducible gene has been reported to direct the synthesis of an endogenous siRNA that plays a role in *Craterostigma* dehydration tolerance [115]. Northern blot analysis in wheat seedlings indicated that the expression of four siRNA changed significantly in response to cold, heat, salt, and dehydration stresses [116], (Table 1). Heat and NaCl stress suppressed the expression of 002061_0636_3054.1 siRNA and 005047_0654_1904.1 siRNA [116]. The genes targeted by these siRNAs may be significant in regulating multiple stress responses in wheat. 

In *Arabidopsis*, transgenerational responses are based on DNA methylation mediated by hcsiRNAs during abiotic stress [117]. Moreover, modification triggered by histone deacetylases 2C (HD2C) and HDA6 contributes to water deficit and ABA responses [118,119]. Loss of HDA6 reactivated transcription of an RdDM target [120], while HDA6 and HD2C interacted with DNA methyltransferase MET1 and AtDNMT2, respectively [121,122]. Collectively, the association between histone deacetylases and DNA methyltransferases indicates that HDA6 and HD2C are part of sncRNA regulatory pathway that is involved in water deficit and ABA responses.

In other studies, ta-siRNA TAS3a-5′D6 (+) was reported to regulate the auxin signaling pathway by guiding the cleavage of ARF, thereby playing a key role in the adaptation of wheat to cold stress [123]. Similarly, three TAS3 derived ta-siRNAs were shown to target cassava ARFs under cold stress conditions [124], (Table 1). Thus, ta-siRNA ‘s response to cold stress is correlated with auxin signaling in plants. In cassava, two nat-siRNAs were differentially expressed in response to cold stress. One of them encoded the transcriptional regulator of the NAC (No Apical Meristem) domain, which has been involved in plant response to cold [124].

### 4.3. Roles of lncRNAs in Plant Abiotic Stress Response

Recent studies have shown that plant lncRNAs are involved in various abiotic stress responses [80,125]. These lncRNAs have been reported to execute their functions in responding to abiotic stresses in different ways. First, some plant lncRNAs participate in abiotic stress response through target mimicry. They act as competitive endogenous RNAs (ceRNAs) targeted by miRNAs, thereby blocking miRNA interactions with their targets [126]. In *Arabidopsis* under P deficiency conditions, lncRNA IPS1 was reported to mimic miRNA399, thereby blocking it from interacting with its target PHOSPHATE 2 (PHO2), resulting in weakened miR399- mediated repression of PHO2 [73,74,127], (Table 2). Similar mimicry roles of lncRNAs have been reported in rice under P deficiency [128]. In *Populus trichocarpa*, ptc-miR482a.1, ptc-miR476a, and ptc-miR156c were reported as targets mimicry of drought-responsive lncRNAs [129] (Table 2).

Plant lncRNAs can act as sRNA precursors during abiotic stress response [70]. Nine lincRNAs serving as precursors for 11 known miRNAs were identified in a study of *Populus* under N deficiency, while another five lincRNAs were precursors for 14 novel miRNAs [130]. In his study, Song et al. [131] reported 9,687 novel lncRNAs and 50 lncRNAs, which acted as miRNA precursors in *Brassica rapa* under cold and heat stress. Similar results were reported in *Phaeodactylum tricornutum* under P deficiency conditions [132], (Table 2). In *Populus simonii*, two lncRNAs, Psi-lncRNA00020674 and Psi-lnc00201294 were suggested to be the precursors of miRNA166a and miRNA27 by overlapping with the genomic regions generating these miRNAs [122]. Several lncRNAs were identified as miRNA, siRNA, and shRNA in *Zea mays* under drought stress conditions [133], and wheat (*Triticum aestivum*) under heat stress [116,134], (Table 2).

Plant antisense lncRNAs interact with sense mRNAs during abiotic stress response. Such interactions lead to the formation of double-stranded RNA duplexes, thus affecting the expression of the gene on the opposite strand [135]. Five antisense lncRNAs were found in poplar under nitrogen deficiency conditions [131]. Similar findings have also been reported in maize under drought stress [133] and in *Arabidopsis* under light and heat stress conditions [135,136] (Table 2).

Also, some plant lncRNAs are responsive to abiotic stress through chromatin remodeling [78]. The expression of COOLAIR and COLDAIR represses FLC in cold-stressed *Arabidopsis* via lncRNA-mediated chromatin modifications (lncR2Epi) [137,138], (Table 2). COOLAIR mediates the reduction of H3K36me3 or H3K4me2 at FLC [137,138] while COLDAIR combines with polycomb repressive complex2 (PRC2) to promote H3K27me3 accumulation at FLC [137,138]. In *Arabidopsis*, the expression of 40–50% of light-responsive sense/antisense transcripts associated with H3K9ac and/or H3K27ac were significantly regulated under high light stress conditions [135]. Therefore, histone modification and histone acetylation are crucial for the light-responsive expression changes of NATs.

Some lncRNAs respond to environmental stress through the RdDM pathway. In tomatoes, suppression of SlAGO4A, which codes for a core factor of the RdDM pathway, significantly increased resistance of salt and drought stress compared to wild species and transgenic plants over-expressing SlAGO4A [139]. In response to heat stress, the *Arabidopsis* mutants *rdr2* and *dcl3* exhibited reduced survival rates [140]. In *Arabidopsis*, heat stress-induced the expression of a vacuolar protein 4 (Vps4) gene with an enhanced expression of the same gene observed in the *nrpd2* mutant [140]. The RdDM pathway regulated an MYB domain protein 74 (MYB74) in *Arabidopsis* during salt stress conditions [141].

## 5. Conclusions and Future Perspectives

Advancements in high-throughput sequencing technology have provided a vital understanding of the fundamental roles that ncRNAs perform during abiotic stress response in plants. The complexity of the ncRNA pathways, however, parallels the diverse spectrum of eukaryotic regulatory pathways, and dozens of questions pertaining to ncRNA functions still need to be addressed.

Despite having a common origin and being conserved in different plants, some ncRNAs exhibit discrete expression patterns in responses to the same abiotic stress across plant species. Moreover, some ncRNAs exhibit tissue-specific expression while others are responsive to specific environmental stresses. Such findings suggest that ncRNA expression is regulated at the level of transcription. However, an additional in-depth research is required to unravel how ncRNA expression is regulated at the transcriptional level in response to environmental changes and whether other transcripts and protein-protein interactions involved in the biogenesis of such ncRNAs are themselves tissue- and time-specifically expressed.

At a molecular level, ncRNAs are controlled through multiple ways, including transcription and post-transcription. However, how plants synchronize these processes to regulate the buildup of ncRNAs in response to abiotic stress remains poorly studied. Furthermore, plant response to abiotic stresses requires the coordinated action of various pathways. It is poorly understood how ncRNAs coordinate with other pathway networks to regulate different physiological processes during stress conditions.

Plants ncRNAs vary widely from each other, but they communicate in their mechanisms of action. Their coordinated response suggests that at some stage, these distinct molecules are interconnected. Interestingly, during the environmental stress response, they function collectively as well as individually. However, the functions of most ncRNAs are still unclear, and there is a significant gap between the discovery of ncRNA genes and the verification of their functions. The discovery of more ncRNAs will simultaneously provide researchers with the ability to exploit these ncRNAs in favor of generating plants that can survive abiotic stress. If the regulatory breadth of ncRNAs is as anticipated, then these molecules will be instrumental in improving plant yields, quality, and resistance to various environmental stresses.

## Figures and Tables

**Table 1:** Examples of siRNAs associated with Abiotic stress response in plants.

siRNA	Abiotic Stresses Regulated	Plant Species	References
SRO5-P5CDH nat-siRNA	↓ by salt stress	*Arabidopsis*	[34]
TAS1, TAS2, TAS3 ta-siRNA	↑ by hypoxia stress	*Arabidopsis*	[103,104,105]
HTT1, HTT2-TAS1	↑ by heat stress	*Arabidopsis*	[106,107]
TAS4 ta-siRNAs	↑ by phosphate deficiency stress	*Arabidopsis*	[108,109]
TAS4-siR81(-)	↑ by nitrogen deficiency stress	*Arabidopsis*	[109]
hcsiRNAs (ONSEN)	↑ by heat stress	*Arabidopsis*	[110,111,112]
nat-siRNAs	↑ by heat stress	Cabbage	[113,114]
nat-siRNAs	↑ & ↓ by heat stress	*Brassica rapa*	[114]
CDT1-siRNA	↑ by dehydration stress	*Craterostigma*	[115]
002061_0636_3054.1 siRNA	↓ by heat, NaCl, & dehydration	Wheat	[116]
005047_0654_1904.1 siRNA	↓ by heat, NaCl, & dehydration	Wheat	[116]
005047_0654_1904.1 siRNA	↑ by cold stress	Wheat	[116]
080621_1340_ 0098.1 siRNA	↑ by cold stress but ↓ by heat stress	Wheat	[116]
007927_0100_2975.1 siRNA	↓ by cold, NaCl, and dehydration	Wheat	[116]
hcsiRNAs (HD2C, HDA6)	↑ & ↓ by drought, ABA stress	*Arabidopsis*	[117,118,119,120,121,122]
ta-siRNA TAS3a-5′D6 (+)	↑ by cold stress	Wheat	[123]
3 ta-siRNAs	↑ & ↓ by cold stress	Cassava	[124]
2 nat-siRNA	↑ & ↓ by cold stress	Cassava	[124]

The symbol ↑, an upward pointing arrow refers to induced expression while the arrow symbol ↓, a downwards pointing arrow refers to suppressed expression of siRNAs during corresponding abiotic stress.

**Table 2:** Examples of lncRNAs associated with abiotic stress response in plants.

lncRNAs	Mechanism	Stress Association	References
IPS1	miR399 target mimicry	↑ in response to phosphate deficiency (*Arabidopsis thaliana*)	[73,74,127]
lncRNAs	target mimicry	↑ & ↓ in response to phosphate deficiency (*Oryza sativa*)	[128]
lincRNA1128	ptc-miR482a.1 target mimicry	↓ in response to drought stress (*Populus trichocarpa*)	[129]
lincRNA1393	ptc-miR6459b target mimicry	↓ in response to drought stress (*Populus trichocarpa*)	[129]
lincRNA3018	ptc-miR399i target mimicry	↓ in response to drought stress (*Populus trichocarpa*)	[129]
lincRNA2752	ptc-miR169o target mimicry	↑ in response to drought stress (*Populus trichocarpa*)	[129]
lincRNA1795	ptc-miR476a target mimicry	↓ in response to drought stress (*Populus trichocarpa*)	[129]
lincRNA20	ptc-miR476a target mimicry	↑ in response to drought stress (*Populus trichocarpa*)	[129]
lincRNA2623	ptc-miR156k target mimicry	↓ in response to drought stress (*Populus trichocarpa*)	[129]
lincRNA2623	ptc-miR156c target mimicry	↓ in response to drought stress (*Populus trichocarpa*)	[129]
lincRNA967	ptc-miR6462e target mimicry	| in response to drought stress (*Populus trichocarpa*)	[129]
lincRNA2762	ptc-miR156k target mimicry	↓ in response to drought stress (*Populus trichocarpa*)	[129]
lincRNA1449	ptc-miR156k target mimicry	| in response to drought stress (*Populus trichocarpa*)	[129]
lincRNA179	ptc-miR156a target mimicry	| in response to drought stress (*Populus trichocarpa*)	[129]
lincRNA2198	nd	↑ in response to drought stress (*Populus trichocarpa*)	[129]
lincRNA2131	nd	↑ in response to drought stress (*Populus trichocarpa*)	[129]
lincRNA2085	nd	↑ in response to drought stress (*Populus trichocarpa*)	[129]
lincRNA2962	nd	↑ in response to drought stress (*Populus trichocarpa*)	[129]
lincRNA1534	nd	↑ in response to drought stress (*Populus trichocarpa*)	[129]
lincRNA1039	nd	↑ in response to drought stress (*Populus trichocarpa*)	[129]
lincRNA2962	nd	↓ in response to drought stress (*Populus trichocarpa*)	[129]
lincRNAs	miRNAs precursors	↑ & ↓ in response to nitrogen deficiency stress (*Populus tomentosa*)	[130]
lincRNAs	miRNAs precursors	↑ & ↓ in response to cold and heat stress (*Brassica rapa*)	[131]
pti-MIR5472	miR5472 precursors	↑ in response to phosphate deficiency (*Phaeodactylum tricornutum*)	[132]
pti-MIR5471	miR5471 precursors	↑ in response to phosphate deficiency (*Phaeodactylum tricornutum*)	[132]
lncRNAs	sRNA precursors	↑ in response to drought stress (*Zea mays*)	[133]
TalnRNA5	ta-miR2004 precursors	↑ in response to heat stress (*Triticum aestivum*)	[116,134]
TahlnRNA27	ta-miR2010 precursors	↑ in response to heat stress (*Triticum aestivum*)	[116,134]
TalnRNA21	siRNA precursors	↑ in response to heat stress (*Triticum aestivum*)	[116,134]
TahlnRNA3	siRNA precursors	↑ in response to heat stress (*Triticum aestivum*)	[116,134]
TahlnRNA14	siRNA precursors	↑ in response to heat stress (*Triticum aestivum*)	[116,134]
TahlnRNA19	siRNA precursors	↑ in response to heat stress (*Triticum aestivum*)	[116,134]
TahlnRNA36	siRNA precursors	↑ in response to heat stress (*Triticum aestivum*)	[116,134]
TahlnRNA41	siRNA precursors	↑ in response to heat stress (*Triticum aestivum*)	[116,134]
TahlnRNA42	siRNA precursors	↑ in response to heat stress (*Triticum aestivum*)	[116,134]
TahlnRNA47	siRNA precursors	↑ in response to heat stress (*Triticum aestivum*)	[116,134]
TahlnRNA52	siRNA precursors	↑ in response to heat stress (*Triticum aestivum*)	[116,134]
lincRNAs	antisense transcription	↑ & ↓ in response to nitrogen deficiency stress (*Populus tomentosa*)	[130]
lncRNAs	antisense transcription	↑ in response to drought stress (*Zea mays*)	[133]
lncRNAs	antisense transcription	↑ in response to light stress (*Arabidopsis thaliana*)	[135]
asHSFB2a	antisense transcription	↑ in response to heat stress (*Arabidopsis thaliana*)	[136]
COOLAIR	chromatin remodeling	↑ in response to cold stress (*Arabidopsis thaliana*)	[137]
lncRNAs	histone modification	↑ in response to light stress (*Arabidopsis thaliana*)	[135]
COLDAIR	histone modification	↑ in response to cold stress (*Arabidopsis thaliana*)	[138]
lncRNAs	RdDM pathway	↓ in response to salt and drought stress (tomatoes)	[139]
lncRNAs	RdDM pathway	↓ in response to heat stress (*Arabidopsis thaliana*)	[140]
lncRNAs	RdDM pathway	↓ in response to salt stress (*Arabidopsis thaliana*)	[141]

The symbol ↑, an upward pointing arrow refers to induced expression while the arrow symbol ↓, a downwards pointing arrow refers to suppressed expression of lncRNAs during corresponding abiotic stress. Similarly, the symbol |, without any pointing direction refers to non-responsive lncRNAs while nd indicates that the mechanism of that particular lncRNA has not yet been established.

## Abbreviations

AGO	Argonaute protein
DCL	Dicer-like protein
SE	Serrate
HUA	HUA Enhancer
RDRs	RNA-dependent RNA polymerases
HYL1	Hyponastic leaves 1
ncRNA	Non-coding RNA
hcsiRNAs	Heterochromatic siRNAs
miRNA	MicroRNA
sRNA	Small RNAs
lncRNA	Long non-coding RNA
sncRNA	Small non-coding RNA
siRNA	Short interfering RNA
tasiRNA	Trans-acting RNA
RISC	RNA-induced silencing complex
SGS	Suppressor of gene silencing
NGS	Next-generation sequencing
TGS	Transcriptional gene silencing
PTGS	Post-transcriptional gene silencing
RdDM	RNA-directed DNA methylation
ORF	Open reading frame
PEG	Polyethylene glycol
UTR	Untranslated region
FLC	Flowering Locus C
